# Association between estimated pulse wave velocity and all-cause mortality in patients with coronary artery disease: a cohort study from NHANES 2005–2008

**DOI:** 10.1186/s12872-023-03435-0

**Published:** 2023-08-21

**Authors:** Chunwei Chen, Wei Bao, Chengwen Chen, Lingyao Chen, Liudi Wang, Haibin Gong

**Affiliations:** 1 Graduate School of Xuzhou Medical University, Xuzhou, Jiangsu China; 2 XuZhou Clinical School of Xuzhou Medical University, Xuzhou, Jiangsu China; 3 Xuzhou Cardiovascular Disease Institute, Xuzhou, Jiangsu China

**Keywords:** Estimated pulse wave velocity, Coronary artery disease, Risk factors, All cause mortality

## Abstract

**Background:**

Arterial stiffness has been shown to be an independent risk factor for adverse events and all-cause mortality in patients. Although PWV is the gold standard for pulse wave velocity, its application in clinical practice is limited by the high cost and complexity. ePwv is a new, simple, non-invasive indicator of arterial stiffness. The aim of this study was to assess the relationship between ePwv and all-cause mortality in patients with coronary artery disease.

**Methods:**

This is a cohort study, selected from NHANES 2005 to 2008, 402 patients with coronary artery disease were included. The ePWV was divided into two groups and KM survival curves were used to calculate cumulative mortality in patients with coronary artery disease. Restricted cubic spline were used to represent the relationship between ePWV and all-cause mortality in patients with coronary artery disease. Cox regression was used to diagnose the relationship between ePwv and all-cause mortality.

**Results:**

The mean age of the study subjects was 68.5 ± 11.8 years, of which 282 (70.1%) were men and 120 (29.9%) were women. During 180 months of follow-up, 160 all-cause mortality occurred. KM survival curves indicated that all-cause mortality increased with increasing ePWV. The relationship between ePWV and all-cause mortality in patients with coronary artery disease was verified by cox models. Patients in higher ePWV tertile tended to have higher all-cause mortality. After complete multivariate adjustment, an increase in ePWV was positively associated with all-cause mortality (HR = 1.180, 95% confidence interval (CI): 1.056–1.320). The multivariate-adjusted HR and 95% CI for the highest ePWV tertile was 1.582 (95% CI: 0.968–2.587) compared to the lowest tertile. In addition, the association between ePWV and mortality was visualized employing restricted spline curves, in which we found curves indicating a possible threshold for the effect of ePWV on all-cause mortality, with HR less than 1 when ePWV was less than 11.15 m/s; thereafter, there was a tendency for HR to increase with enhanced ePWV. Subgroup analysis showed that the correlation between ePWV and mortality persisted in population subgroups.

**Conclusion:**

Our findings suggest that higher ePWV is associated with increased all-cause mortality in patients with coronary artery disease, particularly when ePWV exceeds 11.15 m/s.

**Supplementary Information:**

The online version contains supplementary material available at 10.1186/s12872-023-03435-0.

## Introduction

Cardiovascular disease, as one of the main causes of mortality in the world today, affects millions of people every year. The most notable of these is coronary artery disease. This is a heart disease caused by atherosclerotic lesions of the coronary artery, ultimately resulting in myocardial ischemia, hypoxia, or necrosis due to stenosis or obstruction of the vascular cavity. Coronary artery disease is a leading cause of morbidity and mortality worldwide and is considered to be a chronic immune-inflammatory and fibroproliferative disorder caused by lipids[1–9].

Studies have shown that arterial stiffness increases in patients with coronary artery disease and that early changes in arterial stiffness are very important for the long-term prognosis of patients with coronary artery disease [10–13]. Although pulse wave velocity (PWV) can be the gold standard for arteriosclerosis, it is both a traditional risk factor and an independent risk factor for adverse cardiovascular events and all-cause mortality [14–18]. However, due to the high cost and complexity in testing, PWV has not been widely used in clinical practice [19, 20].

Estimated pulse wave velocity (ePWV), a new index of response arterial arteriosclerosis, is in good agreement with PWV. However, studies on the relationship between ePWV and all-cause mortality in patients with known coronary artery disease are still limited. And they use mostly linear models while the use of nonlinear models is still limited. Restricted cubic spline can better fit the nonlinear relationship and identify important key points. This exploration of critical points is important for early intervention in patients with coronary artery disease. Therefore, we selected 402 patients with coronary artery disease from NHANES and explored the relationship between ePWV and all-cause mortality in patients with coronary artery disease by calculating ePWV data and survival status at follow-up to 2019, to guide early intervention in patients with coronary artery disease.

## Methods

### Study population

**Fig. 1:**
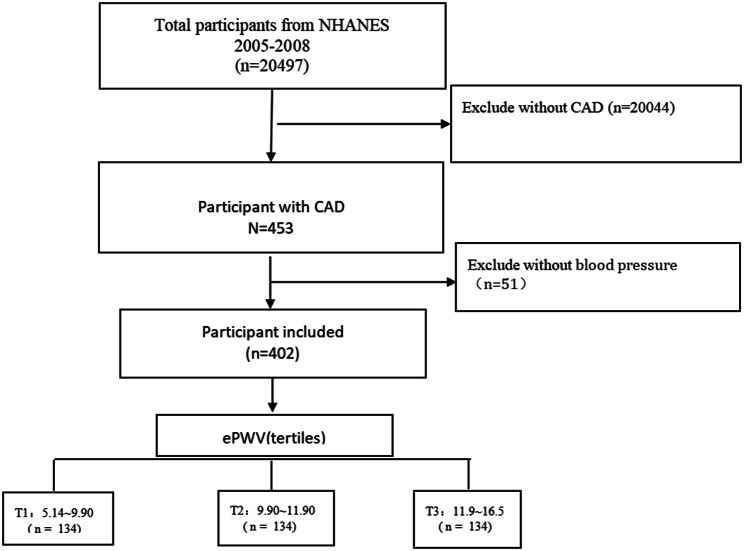
Flow diagram of subjects included in the cohort study

In this study, we analyzed the data from NHANES (n = 20,497) from 2005 to 2008. NHANES estimates the prevalence of major diseases and identifies hazards for the disease by using a complex multi-stage probability sampling design. It aims to gather information about the health and nutrition of the US household population to achieve the goals of disease prevention and health promotion. the official website of NHANES provides data on the design of the study, interview epidemiology, dietary assessment, physical examination, and laboratory tests. The present study sample was limited to patients with coronary artery disease, excluding those without calculating ePWV or with an uncertain presence of coronary artery disease. coronary artery disease diagnosis: doctor or other health experts told you that you have coronary artery disease. There were 453 patients with coronary artery disease, 51 patients with ePWV who could not be calculated. This study includes factors such as gender, age, hypertension, diabetes, Stroke, smoking, drinking, PIR, education level, marital status, BMI, HbA1c, lipids, creatinine, eGFR, ALT, and AST. Ethical consent could be obtained from the CDC, and informed consent has been obtained from all participants. *See* Fig. 1.

### Calculation of ePWV

The ePWV was calculated from age and MBP using the formula obtained from the collaborative reference value of arterial stiffness described by Greve et al. in their study[21, 22]. ePWV = 9.587–0.402age + 4.5610^(-3)age^2–2.62110^(-5)age^2MAP + 3.17610^(-3)ageMAP − 1.83210^(-2)*MAP. Mean BP was calculated as diastolic BP (DBP) + 0.4(SBP − DBP).

### Covariates

After evaluating the relationship between ePWV and the survival status of patients with coronary artery disease, indicators associated with survival status were selected and controlled. The following covariates were collected and considered: age, sex, body mass index, education level, PIR, marital status, HbA1c, smoking, drinking, hypertension, diabetes mellitus, stroke, ALT ,AST ,HDL ,LDL, TC, triglycerides, eGFR,, creatinine. The BMI was calculated by dividing the body weight by the square of the height. Diabetes diagnosed with an FPG equal to or greater than 7 mmol/L or self-reported current use of anti-diabetic drugs. Hypertension was defined based on prior diagnosis, current use of antihypertensive medication, or SBP/DBP 140/90 mmHg. PIR was defined as the family income to poverty line ratio after adjusting for inflation and family size. The selection of covariates was based on previously published studies and available variables.

### Statistical analysis

Categorical variables are reported as frequency and percentage, whereas numerical variables with normally distributed distributions are given as mean ± standard deviation. We used chi-square tests, analysis of variance, or Kruskal-Wallis tests depending on the kind of data to identify variations between individuals in various ePWV tertile. To avoid multicollinearity among independent variables, we considered Variance Inflation Factor (VIF) > 10 as a sign of severe collinearity and excluded the TC indicator. *See supplement 1.*

For the purpose of analyzing the relationship between ePWV and all-cause mortality, we built three COX regression models. To investigate the relationship between ePWV and all-cause mortality, we constructed three Cox regression models. Prior to conducting the statistical analysis, we performed a proportional hazards (PH) test to assess the validity of the proportional hazards assumption for the Cox regression model. (Supplement 2) It is an untuned model, Model 1. In model 2, marital status and gender were adjusted. Gender, BMI, Education, marital status, smoking, drinking, Hypertension, diabetes mellitus, stroke, ALT, AST, HbA1C, creatinine, eGFR, HDL, triglycerides, and LDL are all taken into account in Model 3. In order to evaluate any potential nonlinear link between ePWV and mortality, restricted cubic splines (RCS) were applied.

In addition, we used the maxstat package in R to determine the optimal grouping for ePWV. The maxstat package is a maximum statistic method based on quantiles, which automatically determines the best grouping scheme based on the distribution of the data and the optimal quantile in the population. We used this method to group the ePWV variable and used it for subsequent statistical analysis. *Supplement 3*.

In order to examine the relationship between survival time and survival probability at various ePWV values, we also conducted a Kaplan-Meier survival analysis. The effects of sex, age, hypertension, diabetes, smoking, stroke, PIR, and eGFR on the link between ePWV and mortality were examined using stratified analyses. Statistical significance was defined as a p-value 0.05. R program (version 4.1, Vienna, Austria) and IBM SPSS statistics version 23.0 are required for all statistical studies (Chicago, IL, USA).

## Results

### Characteristics of the study population

**Fig. 2:**
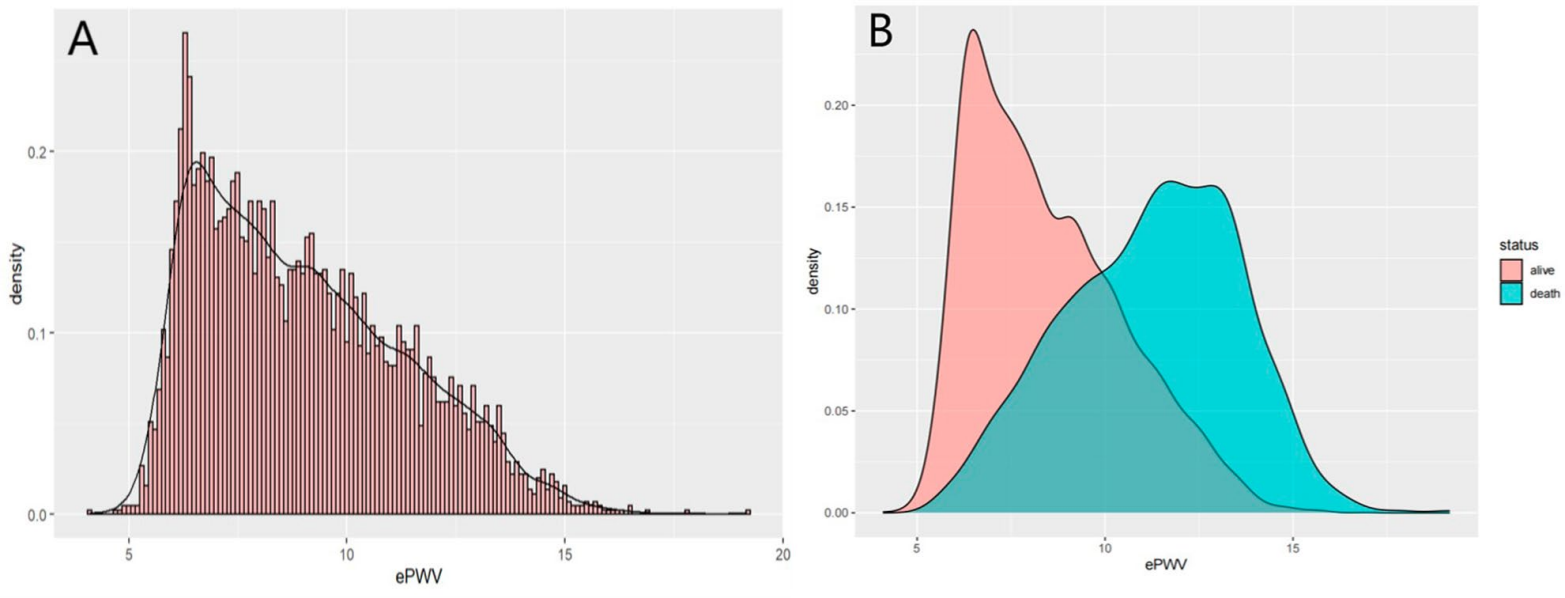
The population distribution of ePWV. (**A**) Density curve shows the distribution of ePWV in the total population. (**B**) The distribution of ePWV in the population with different survival status. ePWV, estimated Pulse Wave Velocity

Table 1 presents the baseline characteristics of the included participants. Almost all characteristics were significantly different across ePWV tertiles except for gender, PIR, education, BMI, DM, HbA1c, AST, and HDL. Participants with higher ePWV (T3) were more likely to be older adults, cigarette smokers, drinkers, and single individuals compared to those with lower ePWV (T1, T2). Additionally, patients with a history of hypertension and stroke were more prevalent among those with higher ePWV. These participants had higher creatinine and LDL levels but lower ALT, eGFR, and triglyceride levels. Furthermore, we observed that participants with higher ePWV tertiles tended to have a higher mortality rate (tiles 1:33.2%, 2:43.7%, 3:67.9%, p < 0.001). The distribution of ePWV in the general population is shown in Fig. 2A, and the stratification of ePWV according to survival status is shown in Fig. 2B. As we can see, the majority of the deceased patients had higher ePWV.

**Table 1:** Baseline characteristics stratified by the estimated Pulse Wave Velocity(ePWV)

*Variables*	*Total(n = 402)*	*T1(n = 134)*	*T2(n = 134)*	*T3(n = 134)*	*p*-Value
Age(year)	68.5 (11.8)	56.6 (9.93)	70.1 (6.85)	78.4 (4.87)	< 0.001
Sex, male, *n* (%)	282 (70.1%)	90 (67.2%)	99 (73.9%)	93 (69.4%)	0.297
PIR	2.41 (1.49)	2.38 (1.63)	2.45 (1.48)	2.41 (1.36)	0.538
**Education level**, *n* (%)					0.389
Less than high school	79 (19.7%)	23 (17.2%)	24 (17.9%)	32 (23.9%)	
High school diploma or GED	72 (17.9%)	28 (20.9%)	23 (17.2%)	21 (15.7%)	
More than high school	251 (62.4%)	83 (61.9%)	87 (64.9%)	81 (60.4%)	
**MS**, *n* (%)					0.005
Married/Living with partner	248 (61.7%)	86 (64.2%)	87 (64.9%)	75 (56.0%)	
Widowed/Divorced/Separated	134 (33.3%)	38 (28.4%)	40 (29.9%)	56 (41.8%)	
Never married	20 (5.0%)	10 (7.5%)	7 (5.2%)	3 (2.2%)	
Stroke, n (%)	65 (16.2%)	14 (10.4%)	21 (15.7%)	30 (22.4%)	0.003
Hypertension, *n* (%)	311 (77.4%)	93 (69.4%)	100 (74.6%)	118 (88.1%)	< 0.001
DM, *n* (%)	175 (43.6%)	60 (45.1%)	62 (46.3%)	53 (39.6%)	0.496
**Smoking**, *n* (%)					< 0.001
Current smoker	68 (16.9%)	37 (27.6%)	24 (17.9%)	7 (5.2%)	
Past smoker	194 (48.3%)	59 (44.0%)	68 (50.7%)	67 (50.0%)	
Never smoker	140 (34.8%)	38 (28.4%)	42 (31.3%)	60 (44.8%)	
**Drinking**, *n* (%)					< 0.001
Former	126 (31.3%)	46 (34.3%)	44 (32.8%)	36 (26.9%)	
Mild	146 (36.3%)	41 (30.6%)	49 (36.6%)	56 (41.8%)	
Moderate	23 (5.7%)	11 (8.2%)	9 (6.7%)	3 (2.2%)	
Heavy	30 (7.5%)	19 (14.2%)	8 (6.0%)	3 (2.2%)	
Never	58 (14.4%)	13 (9.7%)	20 (14.9%)	25 (18.7%)	
BMI	29.9(6.42)	30.1(6.19)	29.7(6.06)	29.9(6.98)	0.806
HbA1c, %	6.18 (1.24)	6.32 (1.55)	6.15 (0.922)	6.03 (1.06)	0.204
ALT, U/L	25.5 (41.4)	25.9 (11.2)	29.5 (71.5)	21.0 (8.56)	< 0.001
AST, U/L	26.6 (20.5)	25.5 (7.16)	28.7 (34.1)	25.4 (7.46)	0.805
Creatinine, mg/dL	1.17 (0.588)	1.12 (0.807)	1.12 (0.339)	1.22 (0.358)	< 0.001
eGFR, mL/min/1.73 m²	69.3 (22.5)	81.0 (24.3)	68.9 (18.3)	58.3 (16.6)	< 0.001
Triglycerides, mg/dL	175 (127)	197 (157)	166 (113)	160 (101)	0.015
Total cholesterol, mg/dL	175 (48.2)	185 (59.6)	167 (38.7)	174 (43.4)	0.023
HDL, mg/dL	47.6 (13.1)	46.5 (14.3)	47.3 (12.0)	49.0 (12.7)	0.062
LDL, mg/dL	93.4 (36.5)	93.7 (33.7)	85.9 (31.9)	101 (42.5)	0.024
Death, %	160 (39.8%)	39 (29.1%)	44 (32.8%)	77 (57.5%)	< 0.001

Values are given as mean ± standard deviation or numbers and percentages. ePWV: T1: 5.14-9.90 m/s,T2:9.90-11.9 m/s, T3:11.9-16.5 m/s; PIR, poverty income ratio; MS, marital status; HbA1c, glycosylated hemoglobin; ALT, alanine transaminase; AST, aspartate transaminase; eGFR, estimated glomerular filtration rate; HDL, high-density lipoprotein; LDL, low-density lipoprotein;DM, diabetes mellitus

### Association between ePWV and death

The results of the univariate and multivariate COX regression analysis are presented in Table 2. The results of the Bonferroni correction can be seen in the supplementary material(Supplement 4). When ePWV was considered as a continuous variable, the unadjusted COX regression model showed that an increase in ePWV seemed to lead to a higher risk of mortality, but this association was not significant (hazard ratio (HR) = 1.045, 95% confidence interval (CI): 0.968–1.128). The association in Model 2 and Model 3 has statistical significance. Participants in the second and third tertiles had a higher risk of mortality in all three models when ePWV was used as a tertile-based categorical variable, with the first tertile serving as a reference. In particular, the risk of mortality was significantly higher in patients in the third tertile.

**Fig. 3:**
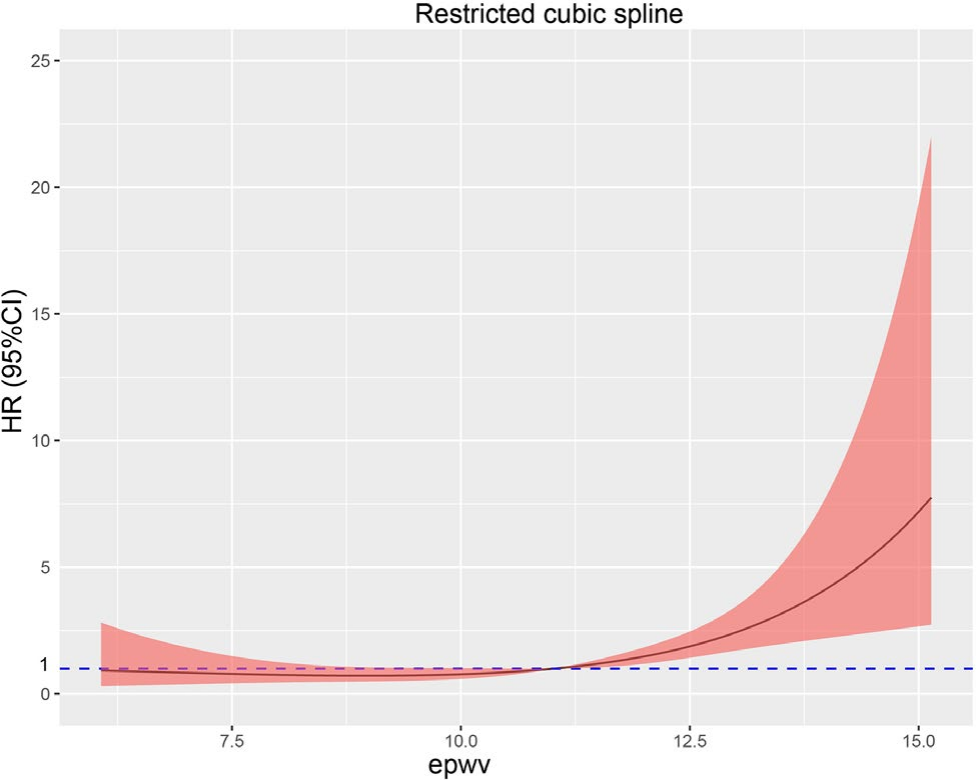
Restricted spline curve shows the relationship between ePWV and Death. Red line and red transparent area represent HR and 95% CI, respectively. HRs (95% CI) were adjusted based on Model 3. ePWV, estimated Pulse Wave Velocity

**Fig. 4:**
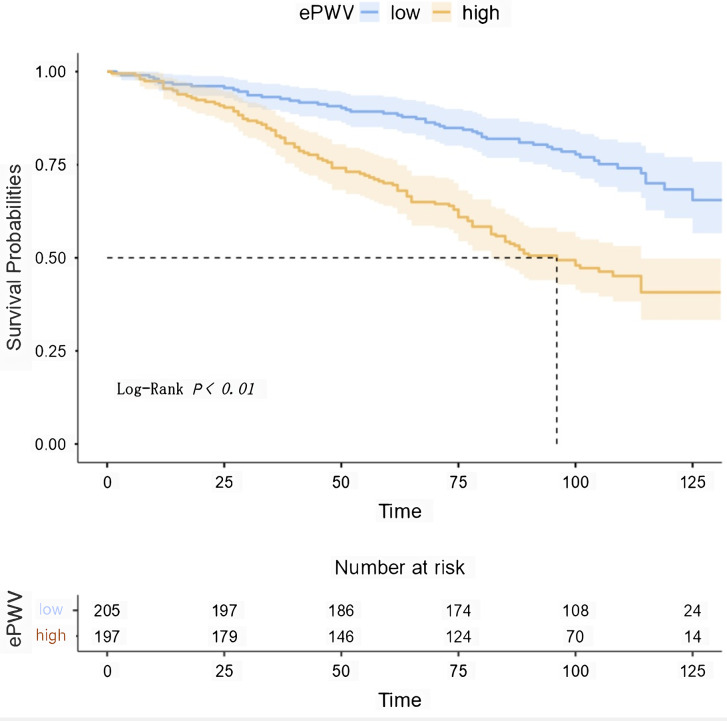
Kaplan-Meier survival curves for patients with different degrees of ePWV. ePWV, estimated Pulse Wave Velocity. low group represents the range of ePWV less than 11.15 m/s, while high group represents the range of ePWV greater than 11.15 m/s

This study used a restricted cubic spline plot and found a non-linear relationship between estimated pulse wave velocity (ePWV) and mortality (Fig. 3). Additionally, we calculated the inflection point to be 11.15 m/s using the maxstat package and ROC curve*( see supplement 3)*. On the curve, we found that the hazard ratio (HR) was less than 1 when ePWV was relatively low (less than 11.15 m/s ). However, the HR tended to increase with an increase in ePWV. We also used Kaplan-Meier survival analysis to evaluate the correlation between survival time and survival probability at different ePWV levels. As shown in Fig. 4, higher ePWV was associated with a higher mortality rate compared to lower ePWV (Log-rank, p<0.01).

**Table 2:** Hazard Ratios (HR) and 95% confidence interval (CI) of the ePWV tertiles for Death

ePWV	Model 1		Model 2		Model 3	
	** h (95% CI)**	**p**	**HR (95% CI)**	**p**	**HR (95% CI)**	**p**
Continuous	1.045(0.968,1.128) 0.2611		1.248 (1.149, 1.356) 0.253		1.180 (1.056, 1.320) 0.0036	
Categorical						
T1 (5.14–9.9)	Ref		Ref		Ref	
T2 (9.90–11.9)	1.343(0.883,2.043) 0.1677		1.350(0.887,2.055) 0.1610		0.857 (0.528, 1.390)0.1309	
T3 (11.9–16.5)	1.438(1.009,2.050) 0.0447		1.436(1.007,2.047) 0.0458		1.582(0.968, 2.587)0.0674	
p for trend	0.0467		0.0479		0.0320	

Data are presented as odds ratios, 95% CIs (confidence intervals), and p-value. Model 1 adjusted for none. Model 2 adjusted for Sex、Marital status, smoking and drinking. Model 3 adjusted for all covariates expect for age. ePWV, estimated Pulse Wave Velocity.

### Stratification analysis

**Fig. 5:**
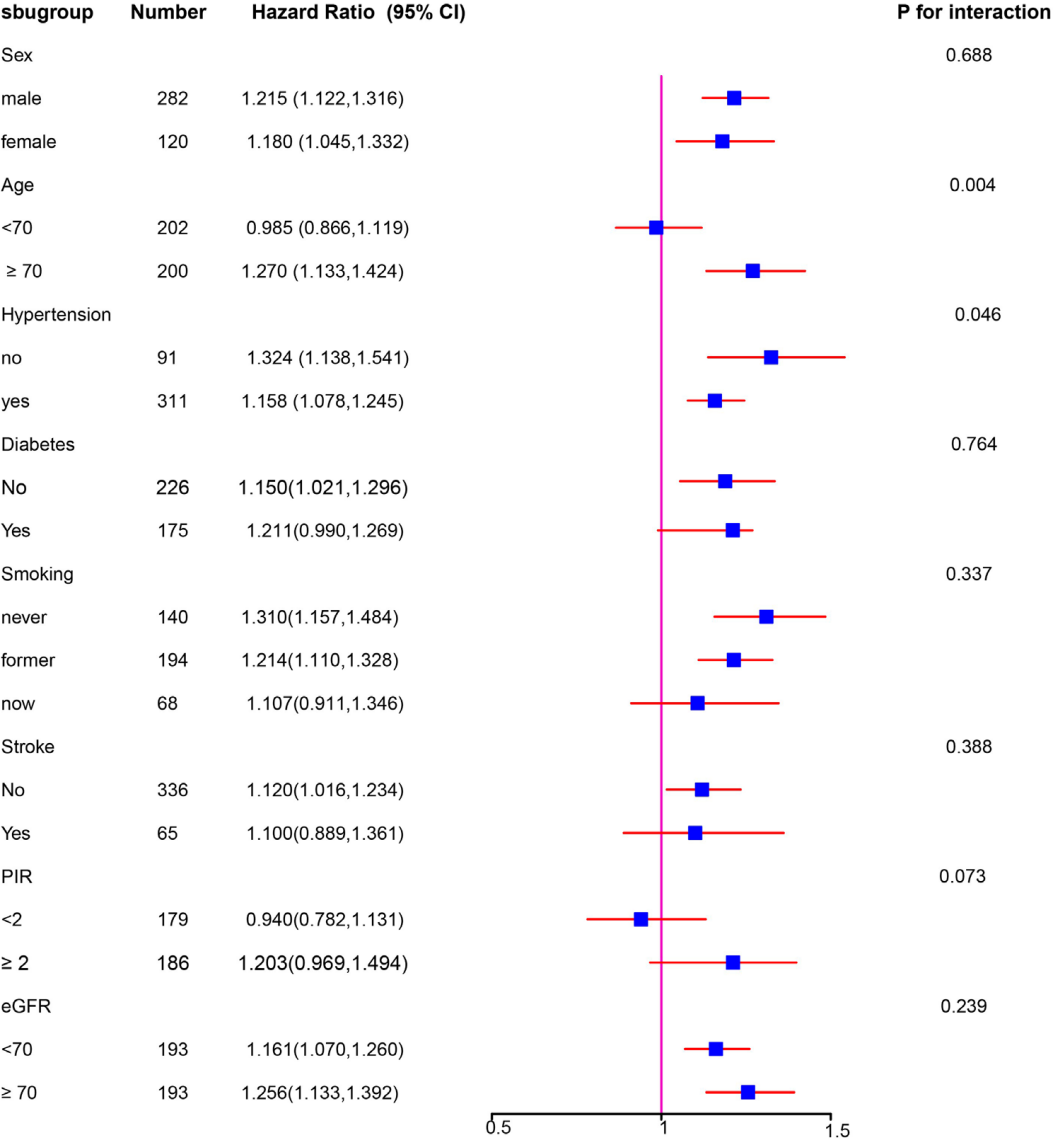
Subgroup analysis of association of ePWV with death. The results were adjusted for sex, age, hypertension, diabetes, smoking, stroke, PIR, and eGFR. PIR, poverty income ratio; eGFR, estimated glomerular filtration rate; ePWV, estimated Pulse Wave Velocity

We conducted subgroup analyses based on characteristics such as sex, age, hypertension, diabetes, smoking, stroke, PIR, and eGFR. In these analyses, ePWV was treated as a continuous variable. We found a statistically significant positive correlation between ePWV and mortality in individuals over 70 (HR = 1.270, 95% CI: 1.133–1.424) and in those without hypertension (HR = 1.324, 95% CI: 1.138–1.541), as illustrated in the forest plot (*See* Fig. 5).

## Discussion

Our study has found a positive association between ePWV and all-cause mortality, which is consistent with previous reports [23–27]. By conducting a sensitivity analysis grouping ePWV into three levels, we found that as ePWV increased, the all-cause mortality predominance ratio showed a statistically significant positive association with p for trend < 0.05. Moreover, restricted cubic splines have visualized the relationship between ePWV and all-cause mortality. Interestingly, we discovered that the risk of mortality did not increase in the curve until ePWV reached about 11.15 m /s. This finding suggests that there might be a threshold for the effect of ePWV patterns on all-cause mortality. The risk of mortality is relatively low when ePWV is less than 11.15 m/s. However, when patients had higher ePWV levels, the risk of all-cause mortality increased significantly. Therefore, we need to pay more attention to patients with coronary artery disease with an ePWV of more than 11.15 m/s to reduce the burden of all-cause mortality.

Our study has also shown that patient risk of mortality was positively associated with ePWV, and the subgroup analysis revealed that ePWV was associated with all-cause mortality at different levels. Most of the patients with higher ePWV observed in this study had higher age and a history of hypertension, which is related to the formula we used to calculate ePWV [28]. Moreover, our study has found that nonsmokers were overrepresented in the population with higher ePWV, which is related to the fact that our study population was a coronary artery disease population. In addition, the high ePWV group had poorer indicators of renal function and higher lipid levels, factors that may also increase the risk of all-cause mortality.

Our study is consistent with the findings reported by Po-ChaoHsu et al. [29–31], who included 881 Taiwanese patients with a median follow-up of 94 months and evaluated ePWV and baPWV as independent predictors of cardiovascular prognosis and all-cause mortality. However, our analysis differs significantly from their study in that their study focused more on the assessment of the cumulative predictive value of ePWV, whereas our work focused on the predictive value of ePWV for all-cause disease and the close relationship between them. Both studies argued that ePWV could be used as a predictor of all-cause mortality in coronary artery disease, and our findings are in line with Esben Laugesen [32], who argued that ePWV can be used as an independent predictor of all-cause mortality in coronary artery disease.

Despite the important findings of our study, there are still some limitations that need to be addressed. First, our analysis can only predict the association between ePWV and all-cause mortality of coronary artery disease, and the causality of this association needs to be verified with more prospective data. This cohort study primarily focuses on patients with coronary heart disease, and analyzes all-cause mortality as the primary outcome. In subsequent studies, the competitive risk model can be used to investigate the competing relationship between cardiovascular death and all-cause mortality. Second, our study was designed to assess fatal events and therefore did not examine the effect of ePWV on nonfatal MACE events. Third, our data were obtained from the NHANES database of adult Americans, and further research is needed to assess whether the results of this study are applicable to other populations with different economic and geographical statuses. Finally, although we adjusted for relevant confounders, there are still possible relevant potential confounders that were not adjusted for. Therefore, studies with larger sample sizes and more variables are needed to confirm the association between ePWV and all-cause mortality from coronary artery disease.

## Conclusions

To sum up, patients with coronary artery disease who undergo long-term follow-up show a significant association between ePWV and all-cause mortality. The risk of all-cause mortality increases considerably when ePWV is higher than 11.15 m/s. Given this, it is crucial to adjust the risk assessment and management plan based on ePWV when managing patients with coronary artery disease.

### Electronic supplementary material

Below is the link to the electronic supplementary material.

Additional File: Supplementary file

## Data Availability

The data of this study are public data and can be obtained on the official website of NHANES : https://www.cdc.gov/nchs/nhanes.
