# Supplementary material for: Association between estimated pulse wave velocity and all-cause mortality in patients with coronary artery disease: a cohort study from NHANES 2005–2008
Source: BMC Cardiovasc Disord. 2023 Aug 21;23:412. doi: 10.1186/s12872-023-03435-0 (PMC10441734; doi:10.1186/s12872-023-03435-0)
Supplement: Supplementary file 1 — Additional File: Supplementary file [file 12872_2023_3435_MOESM1_ESM.docx]

|  | Step 1  VIF | Step 2  VIF |
| --- | --- | --- |
| ePWV | 6.2 | 6.1 |
| Age | 6.5 | 6.5 |
| Sex | 1.5 | 1.4 |
| PIR | 1.5 | 1.5 |
| BMI | 2.6 | 2.5 |
| Education level | 1.4 | 1.4 |
| Marital Status | 1.4 | 1.4 |
| Stroke | 1.3 | 1.3 |
| Hypertension | 1.2 | 1.2 |
| DM | 2 | 1.9 |
| eGFR | 4.7 | 4.6 |
| smoke | 3.2 | 3.2 |
| drink | 2.9 | 2.9 |
| HbA1c | 4 | 4 |
| ALT | 3.1 | 3.1 |
| AST | 2.6 | 2.6 |
| Triglycerides | 7.1 | 1.5 |
| Total cholesterol | 48.5 | NA |
| HDL | 6.7 | 1.9 |
| LDL | 32.1 | 1.4 |

**Supplementary Material 1 :**

In this study, two multicollinearity checks were conducted, and a table of variance inflation factors (VIFs) was generated. VIFs greater than 10 were considered as indicating severe collinearity, and thus the TC variable was excluded from the regression model.

**Supplementary Material 2 :**

In the PH (proportional hazards) test, the Schoenfeld residual test is used to assess whether the Cox proportional hazards assumption holds. When there is no significant correlation between the Schoenfeld residuals and time, it indicates that the Cox proportional hazards assumption holds, while when there is a significant correlation between the Schoenfeld residuals and time, it indicates that the Cox proportional hazards assumption does not hold.

The Schoenfeld test compares the residuals of each predictor variable in the Cox regression model with time to assess whether the proportional hazards assumption is met. If the relationship between a predictor variable and time is not constant (i.e., violates the proportional hazards assumption), a significant residual pattern may emerge, indicating that the assumption is not met.

The results of the Schoenfeld test are typically presented as a set of curves that represent the relationship between the residuals of each predictor variable and time. If the curves are flat or nearly flat, it suggests that the proportional hazards assumption is met; if the curves show clear trends or fluctuations, it may indicate that the assumption is not met. In this case, alternative models or methods can be used to adjust or improve the Cox regression model.

In summary, the Schoenfeld test is an important tool for assessing whether the proportional hazards assumption is met in the Cox regression model, which helps to ensure the accuracy and stability of the model results.

|  | chisq | P-value |
| --- | --- | --- |
| ePWV | 1.151 | 0.169 |
| age | 0.346 | 0.364 |
| sex | 0.005 | 0.942 |
| Education | 0 | 0.988 |
| BMI | 4.53 | 0.033 |
| Marital status | 0.004 | 0.952 |
| Hypertension | 1.415 | 0.234 |
| DM | 1.281 | 0.258 |
| Alt | 3.466 | 0.063 |
| Ast | 0.182 | 0.67 |
| eGFR | 1.58 | 0.209 |
| smoke | 1.153 | 0.562 |
| Drink | 7.83 | 0.098 |
| HbA1c | 11.913 | 0.001 |
| Cr | 0.014 | 0.906 |
| Triglycerides | 1.164 | 0.281 |
| HDL | 0.377 | 0.539 |
| LDL | 1.186 | 0.276 |

**
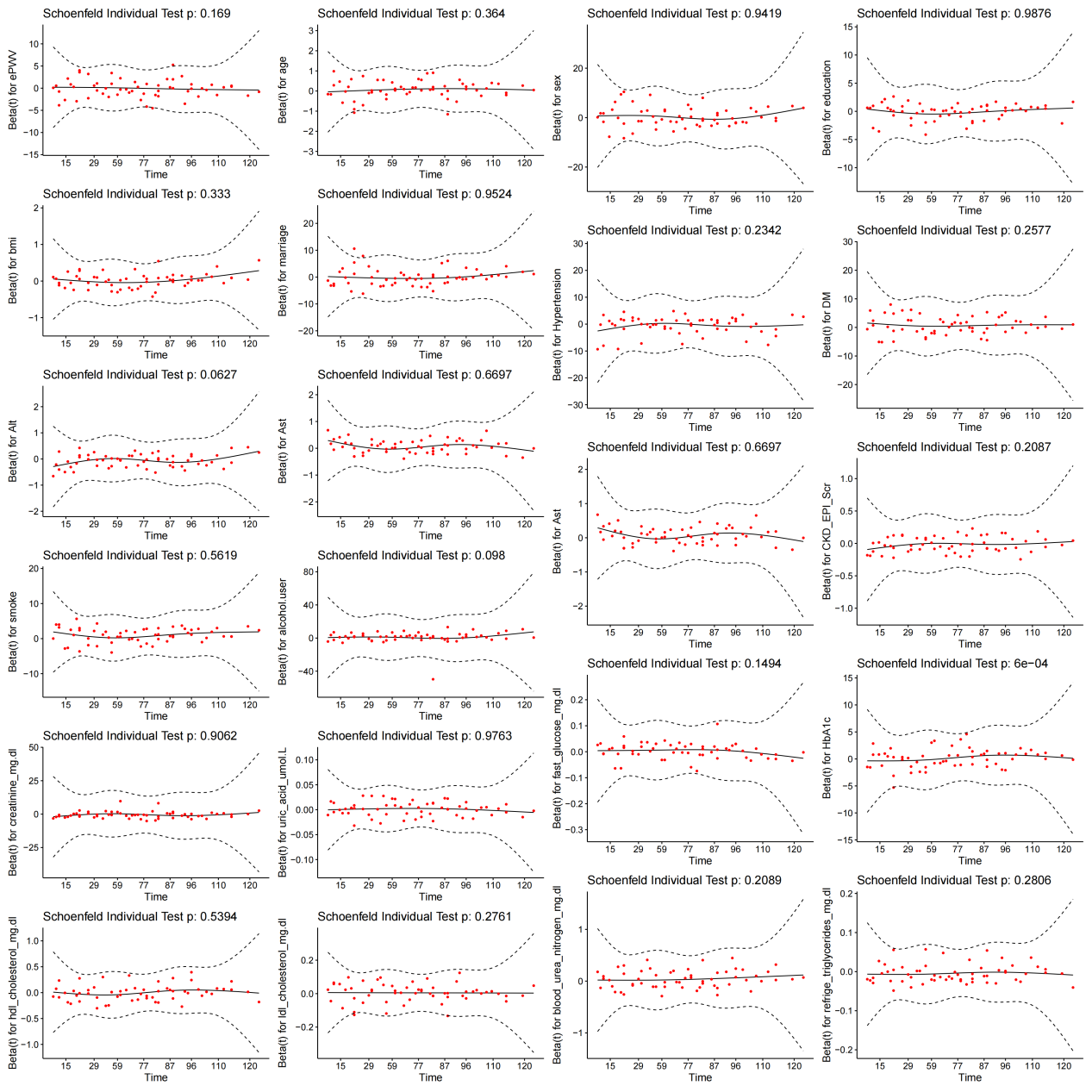
**

**Supplementary Material 3 :**

Both Maxstat and ROC curve analysis methods are commonly used to find the optimal cutoff value, and they have their own advantages and disadvantages. In this study, both methods were used simultaneously and yielded consistent results.


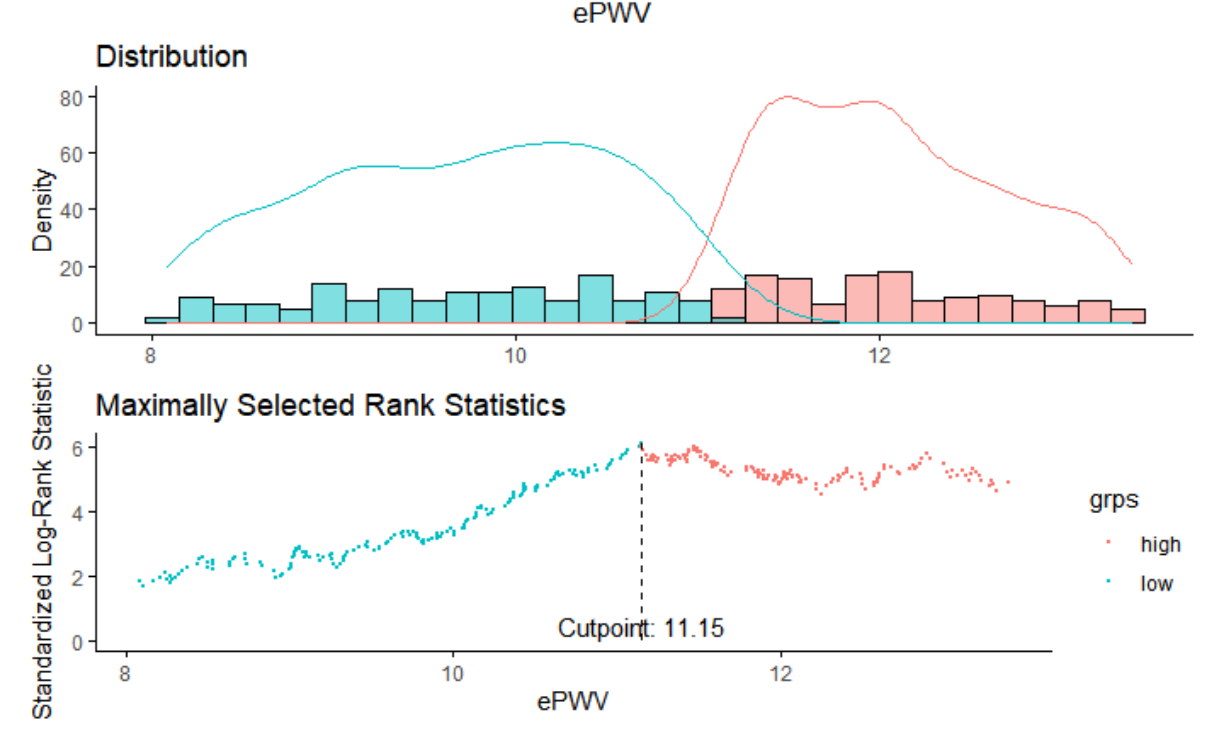


The advantage of Maxstat method is its simplicity and ease of implementation, and it usually yields good results. However, this method only considers the weighted sum of sensitivity and specificity, without considering important indicators such as false positive rate and false negative rate at different cutoff values. Therefore, in some cases, the selected optimal cutoff value may not be accurate or reliable.


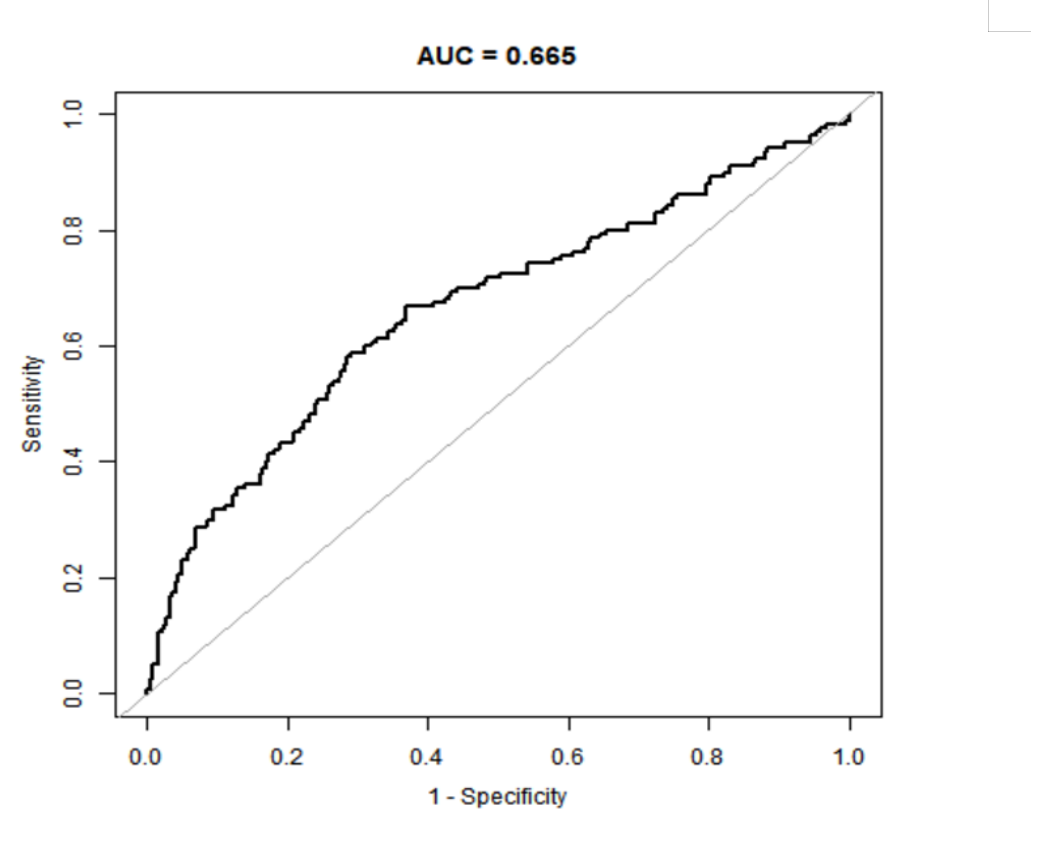


The advantage of ROC curve analysis method is that it can comprehensively consider the trade-off between sensitivity and specificity, and evaluate the overall performance of the classifier by calculating AUC. In addition, this method can adjust for different sample distributions to adapt to different application scenarios. The disadvantage is that it requires more data and computing resources, and a certain technical understanding is needed to interpret the shape of the ROC curve and AUC.

| est | Best threshold | Specificity | Sensitivity | Accuracy | Positive-LR | Negative-LR | Diagnose-OR | N-for-diagnose | Postive-pv | Negative-pv |
| --- | --- | --- | --- | --- | --- | --- | --- | --- | --- | --- |
| ePWV | 11.1515 | 0.6322 | 0.6687 | 0.6468 | 1.8184 | 0.5239 | 3.4706 | 3.3225 | 0.5459 | 0.7427 |

**Supplementary Material 4 :**

Hazard Ratios (HR) and 98.33% confidence interval (CI) of the ePWV tertiles for Death. Adjusted for multiple testing correction using Bonferroni method.

| **ePWV** | **Model 1** | | **Model 2** | | **Model 3** | |
| --- | --- | --- | --- | --- | --- | --- |
|  | **HR (98.33% CI)** | **p** | **HR (98.33% CI)** | **p** | **HR (98.33% CI)** | **p** |
| Categorical | | | | | |  |
| T1 (5.14-9.9) | Ref | | Ref | | Ref | |
| T2 (9.90-11.9) | 1.343（0.273, 2.413） 0.5031 | | 1.350（0.818, 2.119） 0.4830 | | 0.857 （0.556, 1.158） 0.4083 | |
| T3 (11.9-16.5) | 1.438（0.805, 2.071） 0.1341 | | 1.436（0.966, 2.066） 0.1374 | | 1.582（0.968, 2.587） 0.2018 | |
